# Patient acceptance of HIV testing services in rural emergency departments in South Africa

**DOI:** 10.4102/sajhivmed.v21i1.1105

**Published:** 2020-07-22

**Authors:** Aditi Rao, Caitlin Kennedy, Pamela Mda, Thomas C. Quinn, David Stead, Bhakti Hansoti

**Affiliations:** 1 Department of International Health, Johns Hopkins Bloomberg School of Public Health, Baltimore, United States of America; 2 Nelson Mandela Academic Clinical Research Unit, Mthatha, South Africa; 3 Department of Medicine, Johns Hopkins University School of Medicine, Baltimore, United States of America; 4 Division of Intramural Research, National Institutes of Allergy and Infectious Diseases, National Institutes of Health, Bethesda, United States of America; 5 Department of Medicine, Frere and Cecilia Makiwane Hospitals, East London, South Africa; 6 Department of Medicine, Faculty of Health Sciences, Walter Sisulu University, East London, South Africa

**Keywords:** HIV counselling and testing, South Africa, emergency department, patient acceptance, implementation research, linkage to care

## Abstract

**Background:**

South Africa faces the highest burden of HIV infection globally. The National Strategic Plan on HIV recommends provider-initiated HIV counselling and testing (HCT) in all healthcare facilities. However, HIV continues to overwhelm the healthcare system. Emergency department (ED)-based HCT could address unmet testing needs.

**Objectives:**

This study examines the reasons for accepting or declining HCT in South African EDs to inform the development of HCT implementation strategies.

**Method:**

We conducted a prospective observational study in two rural EDs, from June to September 2017. Patients presenting to the ED were systematically approached and offered a point-of-care test in accordance with national guidelines. Patients demographics, presenting compaint, medical history and reasons for accepting/declining testing, were recorded. A pooled analysis is presented.

**Results:**

Across sites, 2074 adult, non-critical patients in the ED were approached; 1880 were enrolled in the study. Of those enrolled, 19.7% had a previously known positive diagnosis, and 80.3% were unaware of their HIV status. Of those unaware, 90% patients accepted and 10% declined testing. The primary reasons for declining testing were ‘does not want to know status’ (37.6%), ‘in too much pain’ (34%) and ‘does not believe they are at risk’ (19.9%).

**Conclusions:**

Despite national guidelines, a high proportion of individuals remain undiagnosed, of which a majority are young men. Our study demonstrated high patient acceptance of ED-based HCT. There is a need for investment and innovation regarding effective pain management and confidential service delivery to address patient barriers. Findings support a routine, non-targeted HCT strategy in EDs.

## Introduction

South Africa (SA) faces the highest burden of HIV infection globally, with 7.7 million people living with HIV (PLWH) and prevalence ranging from 12.6% to 27% across the country.^1,2^ In 2018, SA had 240 000 new HIV infections and 71 000 AIDS-related deaths.^1^ The Joint United Nations Programme on HIV/AIDS (UNAIDS) adopted the ambitious treatment target of 90-90-90, wherein by 2020 90% of all PLWH would know their status, 90% of whom would be receiving sustained antiretroviral therapy (ART), of whom 90% would have achieved viral suppression.^3^ Currently, in SA, an estimated 90% of PLWH know their status, of whom 68% are accessing ART, and 87% of these have achieved viral suppression.^1^ Over the past two decades, numerous steps have been taken by the government to deliver evidence-based interventions focusing on HIV prevention, treatment, and retention. These efforts include the expansion of condom distribution, a national voluntary medical male circumcision program, prevention of mother-to-child transmission, as well as initiatives to increase knowledge and awareness of HIV/AIDS in communities utilising healthcare, in educational infrastructures and social media.^2,4^ However, despite sustained efforts and innovative measures, critical coverage gaps remain, with an estimated 10% of HIV-positive South Africans unaware of their status^5^ and with 15% of new global infections occurring in the country.^2^

The first critical step to meeting the 90-90-90 target is HIV testing. Early detection of undiagnosed HIV infection followed by effective linkage to care and treatment extends life expectancy, improves the quality of life, and reduces HIV transmission.^6^ Since 2015, the South African National Strategic Plan on HIV, sexually transmitted infections and tuberculosis has recommended provider-initiated HIV counselling and testing (HCT) to all persons attending healthcare facilities as a standard component of medical care, including trauma, casualty, and specialty clinics.^6,7^ Nonetheless, the provision of HCT in healthcare facilities is often hindered by the lack of standardised training and by competing clinical care priorities that prohibit effective service delivery.^5,7^ In addition, resources for HCT have largely been directed to primary healthcare centres and antenatal clinics or are focused on high-risk populations such as sex workers, men who have sex with men, injection drug users, and prisoners.^8,9^ As a result, individuals who do not interact with the healthcare system through these channels, such as young men, often miss being tested.

In SA, 90% of the population accesses healthcare through the public sector. For 28%, the emergency department (ED), a setting that provides high-volume care, is their only point of contact.^10^ In the United States of America, the ED is recognised by the Centers for Disease Control and Prevention to be a crucial venue in implementing the national HIV testing strategy.^11^ Seminal studies have not only quantified the burden of HIV infection in EDs but also have been critical to shaping the US national strategy for HIV; they could similarly address unmet testing needs in SA.^11,12,13,14^ In low- and middle-income countries (LMICs), HIV prevalence in EDs may be high, for example, 19% in Papua New Guinea and 50% in Uganda.^15,16^ Provision of HIV testing in the ED could thus be a critical intervention in curbing the epidemic. Its acceptance in acute care settings, however, has not been widely evaluated in sub-Saharan Africa. Studies have primarily focused on rates of acceptance, without exploring the reasons behind patients’ decisions. Ascertaining the perspectives of patients, especially of those who decline testing, enables the identification of barriers to service delivery and the development of effective strategies to increase HIV diagnosis and linkage to care.

In this exploratory observational study, to determine the feasibility of expanding an ED-based HIV testing strategy in SA, we investigated patient perspectives on accepting or declining HCT and quantified the burden of HIV infection in the ED while implementing the nationally recommended HCT programme. This study will assist policymakers and healthcare providers to inform the integration of HCT in the clinical care pathway and optimise HCT service delivery in this venue, resulting in early engagement in care and treatment initiation, ultimately reducing HIV-associated morbidity and mortality.

## Methods

The Walter Sisulu Infectious Diseases Screening in Emergency Departments (WISE) Study was a prospective observational study. HIV counselling and testing was implemented in the EDs of the Nelson Mandela Academic Hospital (NMAH) and the Mthatha Regional Hospital (MRH) in the Eastern Cape Province, from 27 June to 03 September 2017.

### Study site

The study was conducted in Mthatha, a rural town in the South African province of the Eastern Cape, a region that supports 12.6% of the country’s population.^10^ The area faces a disproportionate burden of acute injuries and illnesses with high rates of HIV and tuberculosis.^7^ It is also one of SA’s poorest provinces and is a key priority area for HIV research and capacity building.^7^ Both hospitals are affiliated with the Walter Sisulu University. Nelson Mandela Academic Hospital is a large tertiary-care referral centre with 24-h trauma services, seeing only patients requiring specialty or surgical interventions referred from other district-level facilities. Mthatha Regional Hospital is a district-level facility that provides care to walk-in patients and referrals from adjacent maternal and childcare facilities. The EDs provide 24-h coverage and see 100–150 patients daily from the surrounding 100-km catchment area. Both sites are relatively low-resourced and not equipped with an electronic medical record (EMR) system, patient tracking system or standardised triage processes. Furthermore, there are no providers specialising in emergency medicine at these sites.

### Study population

Patients presenting to the ED who were aged 18 years and older and clinically stable (defined as the South African Triage Scale designation of ‘non-emergent’) were included in the study. Triage scores were assigned by trained study staff, based on the South African Triage Scale (SATS).^17^ Patients younger than 18 years, not able to provide informed consent (i.e. patients with a depressed level of consciousness or mentally altered) or undergoing active resuscitation were excluded.

### Recruitment and sampling

All patients presenting to the ED during the study period who met the inclusion criteria were approached by trained HCT staff, informed of the ongoing study and offered a point-of-care HIV test. Written informed consent was sought for testing and participation in a survey that asked about reasons for accepting or declining the test. Patients with a known HIV-positive diagnosis were asked if they had access to an antiretroviral (ARV) clinic, if they were on regular treatment and whether they were aware of having developed AIDS or being virally suppressed. Data were also collected on patient demographics, presenting complaint, presenting symptoms and past medical history.

HIV counsellors approached all eligible patients in a large waiting room after they underwent initial triage and administrative processes. Patients consenting to the study were escorted to a private room for testing if possible, whereas patients assigned a bed were tested at the bedside with curtains drawn where possible. Given the lack of an EMR or patient tracking system, HCT staff placed a small dot on the folders of all patients who were approached and offered HCT. Every 4 h, the study supervisors audited the folders of patients located within the ED to ensure that all eligible patients had been approached.

Based on recent survey data from the 2017 South African national HIV prevalence study, HIV prevalence amongst South Africans of all ages was estimated at 14%.^2^ Our study aimed to recruit a sample size of 700 patients at each site. This would present a large enough sample to capture the variation in testing preferences in the study setting, allowing us to detect a difference of greater than 5% from the baseline estimate of 14%, assuming a two-sided *α* of 0.05 and 80% power, for a period of 7 weeks at each site.

### Intervention

Patients were offered point-of-care HIV testing following the South African national HIV testing guidelines.^7^ Patients who consented to the test provided a blood sample obtained through a lancet finger prick. Following the recommended testing algorithm, patients were first tested using the Advanced Quality Anti-HIV 1&2 rapid test (InTec Products, Inc., Fujian, China). Non-reactive samples were reported as an HIV-negative result. Reactive samples were confirmed with an HIV 1/2/O Tri-line HIV rapid test (ABON Biopharm, Hangzhou, China). Confirmed reactive samples were reported as an HIV-positive result, and patients were provided with a referral letter to a local ARV clinic. Confirmed non-reactive samples were reported as an indeterminate result, and patients were counselled to repeat the test in 4–6 weeks. Counselling preceded and followed all tests and included education on HIV transmission, prevention, and management. Results were available within 10–15 min of testing, whereas counselling required an additional 10–15 min, depending on the HIV test result.

### Data collection

Ten local research assistants were hired and trained in rapid point-of-care HCT, good clinical practice and data collection, and were familiarised with the study protocol before the start of the study. Research assistants and study staff worked in shifts to ensure 24-h coverage of the ED.

In tandem with offering HIV testing, HCT staff administered a brief survey. Patient responses to questions about their gender, past medical history, mode of arrival, reason for visit, presenting complaint, and symptoms were recorded as pre-determined binary or categorical options, age was recorded as free text, and reasons for accepting or declining testing were captured via pre-determined categorical options derived from the literature or as free text. Data were recorded on case report forms. These forms were scanned and uploaded onto iDatafax (DF/Net Research, Inc., Seattle, WA, USA) by trained study staff. Following validation and cleaning, data were exported into Excel v.16.9 (Microsoft, Inc., Redmond, WA, USA), and then imported into Stata v.14 (StataCorp, TX, USA) for analysis.

The outcome of interest, *declining HCT*, was measured as a binary variable (‘no’ = 0 and ‘yes’ = 1). The independent variables measured were *age* (18–30, 31–50, 51–70, 70+), *sex* (male, female), *presenting complaint* (trauma, medical), *South African triage score* (death, routine visit, urgent, very urgent, emergent), *access to primary care* (yes, no), *past medical history* (hypertension, coronary artery disease, tuberculosis, diabetes, asthma, chronic obstructive pulmonary disorder, cancer), *visit time* (within regular operating hours, 9 am to 5 pm, or out of regular operating hours), *visit reason* (new complaint, return visit, referral), *mode of transport* (self-transport, ambulance, police), *presenting symptoms* (pain, fever) and *disposition* (death, intensive care unit admission, general admission, emergent surgery, transfer, discharge, absconded).

### Data analysis and statistics

Analysis was conducted on patients unaware of their status, to examine the relationship between the outcome of interest and all other independent variables. Chi-square tests were used to explore individual variable associations with declining HCT. Logistic regression analysis was conducted to assess the contribution of each variable to declining HCT. Bivariate analysis was conducted to estimate the association between the outcome and each predictor variable, as well as multivariate analysis to estimate the independent effect of each predictor variable, adjusting for all others. All variables were included in the final model, following checks for collinearity and goodness of fit and performing a best-subsets variable selection. Sub-group analysis was completed on the top reasons for accepting and declining HCT by gender.

A reference level was selected for categorical variables with multiple responses, and other levels were accordingly compared. Associations were assessed using odds ratios (ORs), 95% confidence intervals (CIs) and *p*-values. A *p*-value of ≤ 0.05 was regarded as statistically significant. A pooled analysis of data collected from both sites is presented; no significant differences were observed between the two sites (Table 1).

**TABLE 1:** Characteristics of emergency department patients at Nelson Mandela Academic Hospital and Mthatha Regional Hospital.

Variable	NMAH		MRH		Total	
	*n* = 622†	%	*n* = 1258†	%	*n* = 1880†	%
**Age**						
18–30	290	46.6	504	40.1	794	42.2
31–50	170	27.3	393	31.2	563	29.9
51–70	115	18.5	230	18.3	345	18.4
70+	47	7.6	131	10.4	178	9.5
**Sex**						
Male	375	60.3	539	42.9	914	48.6
Female	247	39.7	719	57.2	966	51.4
**Presenting complaint**						
Medical	363	58.4	914	72.7	1277	68
Trauma	259	41.6	343	27.3	602	32
**SATS**						
Emergency	0	0	0	0	0	0
Very urgent	186	29.9	358	28.5	544	28.9
Urgent	401	64.5	868	69	1269	67.5
Routine	35	5.6	32	2.5	67	3.6
Deceased	0	0	0	0	0	0
**Access to primary care**	544	87.9	1152	92.1	1696	90.7
**HIV counselling and testing**						
Accepted	415	66.7	882	70.1	1279	69
Declined	207	33.3	376	29.9	583	31

NMAH, Nelson Mandela Academic Hospital; MRH, Mthatha Regional Hospital; SATS, South African Triage Scale.

† , Data were missing for some variables; therefore, numbers do not always add up to the total.

### Ethical consideration

The study was approved by the Johns Hopkins University School of Medicine Institutional Review Board (reference number IRB00105801), the Human Research Ethics Committee from the University of Cape Town (MREC reference number 856/2015), the Human Research Committee of Walter Sisulu University (reference number 069/2015) and the Eastern Cape Department of Health. Written consent was obtained from all participants who enrolled in the study and was required for the collection of demographic data, HCT and a follow-up call for newly diagnosed patients, separately.

## Results

A total of 1010 patients presented to the NMAH ED between 27 June and 13 August 2017. Of these, 727 (72%) patients were approached by HCT staff, and 622 (61.6%) were enrolled in the study. A total of 3245 patients presented to the MRH ED between 24 July and 03 September 2017; of these, 1347 (41.5%) patients were approached by HCT staff, and 1258 (38.8%) were enrolled in the study (Table 1).

Across both sites, 2074 patients were approached by the HCT staff, and 1880 (90.6%) were enrolled in the study. Patients enrolled were slightly female predominant (966, 51.4%), with a median age of 33 years (interquartile range [IQR]:24–59). Most patients presented with medical complaints (1278, 67.9%), received a triage designation of ‘urgent’ (1269, 67.5%) and reported having access to primary care services (1696, 90.2%; Table 1).

**FIGURE 1:**
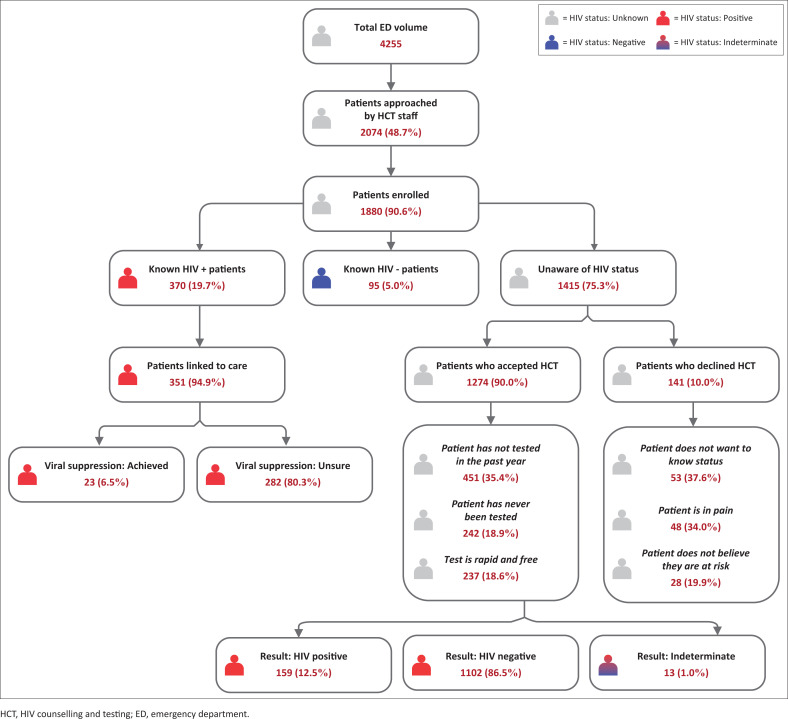
Flow diagram demonstrating study enrolment and outcomes.

Of the 1880 patients enrolled, 465 (24.7%) patients were aware of their HIV status (defined as a known HIV-positive diagnosis [371, 19.7%] or tested HIV negative within the last 12 months [94, 5%]), and 1415 (75.3%) patients were unaware of their HIV status. Of patients with a known HIV-positive diagnosis, 351 (94.9%) said they were regularly accessing an ARV clinic. Of patients who were regularly accessing an ARV clinic, 23 (6.5%) reported being virally suppressed, 46 (13.1%) reported not being virally suppressed and 282 (80.3%) were unsure (Figure 1). In addition, 20 (5.4%) patients who had a known HIV-positive diagnosis wanted to get retested to confirm if they were truly/still HIV positive.

Of the 1415 patients unaware of their status, 141 (10%) declined HCT, and 1274 (90%) accepted. Of the patients who accepted HCT, 159 (12.5%) were diagnosed as HIV positive, 1102 (86.5%) were diagnosed as HIV negative and 13 (1%) had an indeterminate result. The overall prevalence of HIV in the study population was 28.1%. Patients declining and those accepting HCT both largely presented with medical complaints (912, 64.5%), received a triage designation of ‘urgent’ (954, 67.4%), had stated access to primary care services (1255, 89.2%), had no past medical history (929, 65.7%), visited the ED outside of regular hours (799, 56.5%), had a new complaint (878, 62.4%), used self-transport (881, 62.7%), had symptoms of pain (790, 55.8%), had no symptoms of fever (1388, 98.1%), were ultimately discharged from the ED (718, 53.9%) and were aged 18–30 years (629, 44.5%; Table 2).

**TABLE 2:** Characteristics of emergency department patients and association of factors with declining HIV counselling and testing.

Variable	Accepted testing		Declined testing		χ^2^	*df*	*p*‡	Crude		*p*‡	Adjusted		*p*‡
	*n* = 1274†	%	*n* = 141†	%				OR	95% CI		OR	95% CI	
**Age**													
18–30	562	44.1	67	47.5	2.9935	3	0.393	(Ref.)	-	-	(Ref.)	-	-
31–50	305	23.9	34	24.1	-	-	-	0.9	0.6–1.4	0.763	1.1	0.7–1.7	0.795
51–70	254	19.9	20	14.2	-	-	-	0.7	0.4–1.1	0.392	0.8	0.4–1.6	0.589
70+	153	12.0	20	14.2	-	-	-	1.1	0.6–1.9	0.734	1.6	0.8–3.1	0.172
**Sex**													
Male	673	52.8	56	39.7	8.6300	1	0.030*	(Ref.)	-	-	(Ref.)	-	-
Female	601	47.2	85	60.3	-	-	-	1.7	1.2–2.4	0.004*	0.7	0.5–1.1	0.098
**Presenting complaint**													
Medical	835	65.5	77	54.6	6.6215	1	0.010*	(Ref.)	-	-	(Ref.)	-	-
Trauma	439	34.5	64	45.4	-	-	-	1.6	1.1–2.2	0.011*	1.1	0.7–1.7	0.788
**SATS**													
Routine	48	3.8	6	4.3	0.5968	2	0.742	(Ref.)	-	-	(Ref.)	-	-
Urgent	863	67.8	91	64.5	-	-	-	0.8	0.4–2	0.703	0.8	0.3–2.1	0.673
Very urgent	363	28.5	44	31.2	-	-	-	0.9	0.4–2.4	0.947	1.2	0.5–3.2	0.710
**Access to primary care**	1129	88.6	126	89.2	0.3368	1	0.562	1.2	0.7–1.2	0.562	1.3	0.7–2.5	0.358
**Past medical history**	446	35.0	39	27.7	-	-	0.800	0.7	0.5–1	0.081	0.8	0.5–1.4	0.442
**Visit time**													
9 am to 5 pm	553	43.4	62	44	0.0146	1	0.904	(Ref.)	-	-	(Ref.)	-	-
Out of hours	720	56.5	79	56	-	-	-	0.9	0.7–1.4	0.904	0.9	0.6–1.3	0.522
**Visit reason**													
New complaint	800	62.8	78	55.3	3.2318	2	0.199	(Ref.)	-	-	(Ref.)	-	-
Return visit	23	1.8	4	2.8	-	-	-	1.8	0.6–5.3	0.297	1.8	0.6–5.9	0.309
Referral	445	34.9	58	41.1	-	-	-	1.3	0.9–1.9	0.113	1.2	0.8–1.8	0.408
**Transport**													
Self-transport	805	63.2	76	54	4.2602	2	0.119	(Ref.)	-	-	(Ref.)	-	-
Ambulance	453	35.6	62	44	-	-	-	1.4	1.1–2.1	0.040*	1.4	0.9–2.1	0.147
Police	8	0.6	1	0.7	-	-	-	1.3	0.2–10.7	0.793	1.4	1.2–11.4	0.776
**Symptoms**													
Pain	695	54.6	95	67.4	8.4652	1	0.004*	1.7	1.2–2.5	0.004*	1.6	1.0–2.6	0.046*
Fever	23	1.8	4	2.8	0.7217	1	0.396	1.6	0.5–4.7	0.400	1.8	0.6–5.6	0.297
**Disposition**													
Death	4	0.3	0	0	4.2867	6	0.638	(Ref.)	-	-	(Ref.)	-	-
ICU	1	0.1	0	0	-	-	-	1	-	-	1	-	-
Admission	295	23.2	39	27.7	-	-	-	0.4	0–4	0.467	0.2	0–2.4	0.225
Emergent surgery	50	3.9	9	6.4	-	-	-	0.6	0.1–6.2	0.690	0.3	0–2.9	0.288
Transfer	48	3.8	6	4.3	-	-	-	0.7	0.1–6.4	0.718	0.4	0–3.8	0.397
Discharge	492	38.6	49	34.8	-	-	-	0.4	0–3.9	0.447	0.3	0–2.8	0.281
Absconded	1	0.1	1	0.7	-	-	-	1.3	0.1–31.1	0.858	0.7	0–19.1	0.854

OR, odds ratio; CI, confidence interval; ref., reference level; ICU, intensive care unit; SATS, South African Triage Scale.

† , Data were missing for some variables; therefore, numbers do not always add up to the total.

‡ , Associations were tested at the 5% significance level.

* , p < 0.05.,

The top reasons for accepting HCT were ‘has not tested in the past year’ (451, 35.4%), ‘has never been tested’ (242, 18.9%) and ‘test is rapid and free’ (237, 18.6%) (Figure 1). Patients accepting testing were largely male (672, 52.7%). The top reasons for declining HCT were ‘does not want to know status’ (53, 37.6%), ‘in too much pain’ (48, 34%) and ‘does not believe they are at risk’ (28, 19.9%; Figure 1). Patients declining testing were largely female (85, 60.3%).

Sub-group analysis of the reasons for accepting and declining HCT by gender showed slight differences between men and women in the reported reasons (Table 3). The primary reason for accepting HCT for both men and women was ‘has not tested in the past year’, 35.8% and 34.9%, respectively, followed by ‘has never been tested’ (21.4%) for men and ‘test is rapid and free’ (20.3%) for women. The primary reason for declining HCT given by men was ‘does not want to know status’ (66.1%) and ‘in too much pain’ for women (22.4%), followed by ‘in too much pain’ for men (42.9%) and ‘does not believe they are at risk’ (17.6%) for women.

**TABLE 3:** Reasons for accepting or declining HIV counselling and testing by gender.

Reasons	Male patients		Female patients	
	*n*	*%*	*n*	*%*
**Top 3 reasons for accepting HCT**	**673**	**-**	**601**	**-**
Patient has not tested in the past year.	241	35.8	210	34.9
Patient has never been tested.	144	21.4	97	16.1
Test is rapid and free.	115	17.1	122	20.3
**Top 3 reasons for declining HCT**	**56**	**-**	**85**	**-**
Patient does not want to know status.	37	66.1	12	14.1
Patient is in too much pain.	24	42.9	19	22.4
Patient does not believe they are at risk.	13	23.2	15	17.6

HCT, HIV counselling and testing.

Univariate analysis showed that compared with male patients, female patients were more likely to decline HCT Associations were assessed using odds ratio (OR: 1.7; 95%) Confidence Intervals (CI:1.2–2.4). Patients who complained of pain compared with patients who did not (OR: 1.7; 95% CI: 1.2–2.5) and those arriving at the ED by ambulance compared to self-transport or with the police (OR: 1.4; 95% CI: 1.1–2.1) were also more likely to decline HCT. In addition, patients presenting with traumatic injuries compared with medical complaints (OR: 1.6; 95% CI: 1.1–2.2) were more likely to decline HCT. Other factors including age, triage score, access to primary care, past medical history, visit time, visit reason and final disposition did not show a statistically significant correlation with declining HCT (Table 2).

Multivariate analysis showed that patients who complained of pain compared with patients who did not (OR: 1.6; 95% CI: 1.1–2.6) were slightly more likely to decline HCT. Other variables did not show a statistically significant correlation with declining HCT (Table 2).

## Discussion

We found acceptance of HCT services in the ED to be reassuringly high. Our study revealed that 90% of patients who were unaware of their HIV status accepted HIV testing. This was observed despite patients being in a clinical environment where HIV testing is not routinely offered and where patients present with acute injury or illness and are often in pain or moderate distress.^11,18^ These findings are consistent with results from other LMICs. In Kenya and Guyana, it was found that 97.7% and 75.5% of non-critical patients accepted HCT in the ED, respectively.^19,20^ Our results also highlight a substantial burden of undiagnosed HIV: 8.5% of enrolled patients were newly diagnosed as HIV positive. These patients presented to the ED for various clinical complaints, and a positive diagnosis of HIV was an incidental finding.

The two most common reasons for accepting HCT in the ED were ‘has not tested in the past year’, followed by ‘has never been tested’. This positively implies the need for HCT in acute care settings, to cover the existing testing gap, as we are able to capture patients who are currently missed by other testing venues within the healthcare system. Recently, novel approaches for HCT – such as home-based, community-based and couples testing – have been added to traditional facility-based HCT delivery systems, with good acceptance rates.^21,22^ A systematic review of HCT strategies in sub-Saharan Africa reported an acceptance rate of 70% for home-based testing and 76% for community-based testing.^21^ However, despite the variety of testing strategies and venues, a testing gap remains, particularly amongst young adults and men.^23,24^ Considering that 44.1% of all patients accepting testing were young adults (18–30 years) and 52.7% were male, our study demonstrates that the ED is an opportune venue to capture this missed population.

Another factor leading to testing acceptance, reported by a fifth of patients accepting HCT, was ‘the test is rapid and free’. This measure combines both cost and ease/limited time lost to testing. While it is hard to separate the two and determine which is a more significant factor, ensuring that both are addressed is a likely key to maintaining high acceptance of HCT in a fast-moving environment such as the ED. This is supported by a study in Uganda, where 25% of ED patients reported not knowing their HIV status because of the lack of access to free testing services.^16^ At present, HCT services are offered free of cost in all government healthcare facilities in SA; however, maintaining free services can be burdensome for the government, especially if testing services are to be further expanded. Furthermore, ensuring that testing and counselling are not time-consuming and are part of routine clinical care in the ED might address the barrier of having to seek out testing as a discrete task in itself.

Acceptance, however, was not universal. In our study population, women were significantly more likely to decline to test, whereas men were more likely to accept. Similar findings were observed in other studies examining the acceptability of testing in EDs, in both high- and low-resource settings.^20,25^ This might be because women are aware of having access to testing services during antenatal visits, through preventing mother-to-child transmission (PMTCT) programmes or through family planning services, and hence do not need to test in the ED.^1^ This is supported by the finding that more women were already aware of their HIV status; 71.6% of the patients with a known HIV-positive diagnosis were women. In addition, a significant proportion of women presenting to the ED in our context were diagnosed with pregnancy complications or injury wounds, likely justifying ‘in too much pain’ as the primary reason reported for women to decline HCT. On the contrary, though women were more likely to decline HCT, a majority still accepted testing when offered. While it is difficult to generalise individual motivations, factors including lack of social support and fear of stigma or rejection if tested HIV positive, especially in the presence of their partner or family accompanying them to the ED, may otherwise underlie the greater tendency of women to decline HCT.^25^

The top reasons reported for declining HCT in our ED, ‘does not want to know status’ and ‘does not believe they are at risk’, are interestingly established findings from high-income countries and LMICs, across healthcare settings.^16,20,26,27,28,29^ It could be that patients prefer uncertainty rather than facing the psychosocial consequences of an HIV-positive diagnosis, especially considering the imaginable stigma attached to such a diagnosis.^30^ This could be tackled through targeted pre- and post-counselling efforts. On the contrary, it is also possible that the small proportion of patients who declined to be tested are not at risk of contracting HIV and were accurately perceiving their risk. We did not include any risk measures in our survey and are thus unable to precisely indicate individuals who *should* have been tested.

A significant barrier to HCT in the ED and other healthcare facilities frequently described in the literature are stigma and the lack of confidentiality.^20,26,31^ This finding is supported by contextual knowledge, wherein anthropological studies exploring cultural perceptions and practices around HIV in Mthatha have reported pervasive stigma attached to HIV/AIDS, resulting in multiple forms of exclusion based on sexism, racism and homophobia,^30^ The National HIV Prevalence Survey indicated that 26% of people would not be willing to share a meal, 18% were unwilling to sleep in the same room and 6% would not speak to PLWH.^2^ Yet, none of the patients declining testing in our study reported reasons implying real or perceived stigma or the lack of confidentiality. The studies supporting this notion conducted in-depth interviews or had one-on-one conversations with patients, which likely allowed for deeper exploration of patient perspectives on HCT services, whereas given the patient volumes, high turnover and the lack of any coordinated processes in our study setting, it is possible that patients were less likely to report stigma as a reason for declining testing.

Pain was a notable justification for declining testing in this context and showed significant correlation through bivariate and multivariate analysis. The second most common reported reason, ‘in too much pain’, was not surprising, as a high proportion of cases presented with acute traumatic injuries. In addition, traumatic injuries and arriving at the ED in an ambulance, which are critical proxies to pain and the seriousness of a patient’s condition, were positively correlated with declining testing. This finding is specific to declining HCT in the ED and has not been previously reported as a barrier in other testing venues. To address this barrier, the integration of pain management before HCT is recommended. If a patient’s presenting complaint has been addressed by providers, and appropriate action taken, patients might be more likely to accept testing.

Linkage to care is the next critical step following testing. As part of our study, all newly diagnosed patients were counselled extensively on the importance of seeking follow-up care and were given a referral letter. Given that both NMAH and MRH see patients from a 100-km radius, it was challenging to ensure linkage to care, as it would depend on the area individuals came from and the presence and ease of access to an ART clinic. For patients who were local to Mthatha, we were able to direct them to the Gateway Clinic – an ART centre – located within the same campus as the hospital. With the consent of all patients who were newly diagnosed as HIV positive, we collected their names and contact details to conduct follow-up calls after 1 month, 6 months and a year. The follow-up calls will allow us to assess whether individuals have been able to link to care and/or what challenges they are facing in doing so. Results from the follow-up calls will be collated and analysed post-completion. Despite these challenges, 94.9% of patients with a known HIV-positive diagnosis presenting to the EDs reported having access to an ART clinic, and 85.4% of those individuals reported regularly accessing the clinic. These rates are commendable and imply a willingness of patients in this setting to seek follow-up care. However, these are self-reported statistics and could be inflated as a result of social-desirability bias.

Another interesting finding was that a small proportion of patients with a known HIV-positive diagnosis (20, 5.4%) requested a repeat test to confirm their diagnosis. Patients stated that they wanted to confirm whether they were *truly* HIV positive and/or if they were *still* HIV positive. Upon retesting, all 20 patients were HIV positive. The desire to retest when an opportunity presented could likely be a result of mistrust in the healthcare system or a result of the low health literacy in the region, which are both potential barriers to achieving high rates of testing and sustained linkage to care.^30,31^

There are several study limitations to consider. The protocol was to approach every patient presenting in the ED who met the inclusion criteria. However, the lack of infrastructure, systematic medical record-keeping or a patient tracking process made it challenging to retain all patients who presented to the ED. Many patients were missing from the records, whereas others were entered multiple times, making it difficult to keep count of the total number of patients. HIV counselling and testing services were provided 24 h a day, yet we were only able to approach 48% of patients who presented for care. We believe this is, in part, a result of the high volumes of patients and the quick turnaround time, as well as the time-consuming nature of counselling. Patients enrolled in the study may be a biased subset of the ED population, namely, easier to approach, spoke the same language as the HCT counsellors, had milder injuries or conditions and presented at times when the patient volume was lower. Maintaining confidentiality was challenging given the limited space – the EDs in both hospitals were in essence one big room, with beds lined up against each other. Lastly, the study was human-resource intensive. We had a team of four dedicated HCT staff at all times. Nevertheless, greater staff numbers would have allowed the capture of more study subjects. Such a situation would be difficult to sustain in a low-resource setting such as Mthatha.

To optimise our strategy and accurately capture data, given the lack of organisation and clear processes, our data were collected prospectively, whereby we relied less on recorded data and were able to capture most of it in real time. As the ED is busy and sees high patient volumes, we attempted to collect as much data as efficiently as possible, using a survey format with mostly ‘yes’ and ‘no’ questions. However, to have had a better understanding of patient perspectives, the study might have been enhanced by in-depth telephone interviews with a smaller number of patients after they had left the ED.

## Conclusion

Our study demonstrated high patient acceptance of the nationally recommended HCT strategy in an ED setting. The overall adult prevalence of HIV in the ED was high at 28.1%. Patients who were male, young and not in pain or critically injured were more likely to accept HCT, critically supporting the provision of HCT in acute care settings, as it successfully captured an important demographic that has generally been missed through other testing venues. In addition, the lack of significant correlation in demographic or clinical characteristics and HCT uptake argues for a routine, non-targeted strategy in the ED. Our study further reveals the need for continued investment to ensure that HCT is widely available, with provision to effectively identify and manage pain and trauma. Finally, critical to embedding HCT in the routine clinical care offered in the ED will be the confidential conduct of HCT that permits stigma around HIV infection and testing to be appropriately addressed – something that will require further innovation and implementation research.
